# Improvement in the function of self-activating chimeric antigen receptor by replacing the linker sequence

**DOI:** 10.3389/fimmu.2025.1502607

**Published:** 2025-04-16

**Authors:** Taku Kouro, Daisuke Hoshino, Yasunobu Mano, Shoutaro Tsuji, Hidetomo Himuro, Kohzoh Imai, Tetsuro Sasada

**Affiliations:** ^1^ Division of Cancer Immunotherapy, Kanagawa Cancer Center Research Institute, Yokohama, Japan; ^2^ Cancer Vaccine and Immunotherapy Center, Kanagawa Cancer Center, Yokohama, Japan; ^3^ Cancer Biology Division, Kanagawa Cancer Center Research Institute, Yokohama, Japan; ^4^ Department of Medical Technology & Clinical Engineering, Gunma University of Health and Welfare, Maebashi, Japan; ^5^ Kanagawa Cancer Center Research Institute, Yokohama, Japan

**Keywords:** CAR-T, scFv, tonic signaling, T cell exhaustion, cancer immunotherapy

## Abstract

Chimeric antigen receptor (CAR)-T cell therapy is an effective treatment for hematological cancers; however, challenges remain in its application to solid tumors. Among these, the control of CAR-T cell exhaustion is important. The relationship between tonic signals generated by the CAR self-activation and CAR-T cell exhaustion has attracted considerable attention. The magnitude of the tonic signal is known to depend on the structure of the extracellular portion of CAR, but the role of the linker sequence of the single-chain variable region (scFv) in the tonic signal and function in CAR-T cells has not been clarified. In this study, we compared two scFv linkers, G4S and Whitlow/218, in self-activating SKM-CAR, which recognized a malignant mesothelioma-specific modified HEG1 molecule. We observed no differences in cell surface phenotypes, NFAT and NFκB signaling intensities, and gene expression profiles between SKM-CAR T cells with these different linkers. However, switching from the G4S to the Whitlow/218 linker in SKM-CAR-T cells with the CD28 co-stimulatory domain significantly altered cytokine expression after antigen stimulation and improved the *in vitro* tumor cell killing activity, but not the *in vivo* tumor control. This is the first study describing the advantages of the Whitlow/218 linker over the G4S linker for some aspects of CAR-T cell function.

## 1 Introduction

Chimeric antigen receptor (CAR)-T cells are engineered T cells designed to treat cancer and other diseases by expressing chimeric receptors that recognize antigens present on the target cells. To convert antigen recognition into a cellular activation signal, the receptors are equipped with activation domains from receptors such as the CD3ζ chain and co-stimulatory domains from co-stimulatory molecules such as CD28 and 4-1BB. Although designed to be activated upon the recognition of target antigens, several CAR constructs have been found to be spontaneously activated in the absence of antigens, generating tonic signals (1, 2). While the magnitude of the tonic signal depends on the structure of the extracellular portion of the CAR (1), the quality of the tonic signal varies depending on the selection of co-stimulatory domains (3). In the case of SKM-CAR, which recognizes the malignant mesothelioma-specific modified HEG1 molecule, we showed that the tonic signal of SKM-CAR with the CD28 co-stimulatory domain induces the terminal exhaustion of CAR-T cells, whereas that of SKM-CAR with the 4-1BB co-stimulatory domain maintains CAR-T cells in the progenitor exhaustion stage (4). Thus, the selection of the co-stimulatory domain is a measure to avoid the exhaustion of CAR T cells with a strong tonic signal.

However, a fundamental reduction in tonic signals may be a better method to avoid CAR-T cell exhaustion. The tendency of CARs to aggregate on the cell surface depends on the frame region of the immunoglobulin variable region and affects the generation of tonic signal (1). Therefore, grafting the complementarity determining region (CDR) of CARs with high tonic signals onto the frame regions of CARs with low tonic signals may be effective. However, this is not easy because to construct functional CARs, the selection of an appropriate frame region is crucial, using frame regions as similar as possible to the original sequence (5). The single-chain variable fragment (scFv), the antigen recognition unit of the CAR, is composed of variable heavy (VH) and variable light (VL) chains, linked by a short sequence of amino acids called linkers. Currently, most CARs adopt a linker called (G4S)n, a repeat of four glycines and one serine, expecting its high flexibility. In addition, CTL019 (tisagenlecleucel) CAR, which recognizes CD19, uses FMC63 scFv with a different linker called Whitlow/218 (6, 7). Because FMC63 is known to have a very low tonic signal (1), we hypothesized that the Whitlow/218 linker might also contribute to the lower tonic signal. In the present study, we replaced the (G4S)_4_ linker of SKM-CAR with the Whitlow/218 linker to investigate its effects on CAR aggregation, cell surface phenotypes, gene expression profiles, and CAR-T cell functions.

## 2 Materials and methods

### 2.1 Cell lines, mice and mAbs

Phoenix-Ampho packaging cell line (RRID: CVCL_H716) was obtained from ATCC (Manassas, VA, USA). The ACC-MESO-4 malignant mesothelioma cell line (RRID: CVCL_5114) was obtained from the RIKEN Cell Bank (Ibaraki, Japan). The generation of the JurkatΔαβCD8a cell line, a subline of Jurkat (RRID: CVCL_0065) in which the *TCRA* and *TCRB* gene loci have been deleted and which carries a CD8A transgene, has been described elsewhere (8). JurkatΔαβCD8a cells were transfected with NFAT-RE (pGL4.30) or NFκB-RE (pGL4.32) luciferase reporter plasmid (Promega, Madison, WI, USA) using a NEPA21 electroporator (NEPAGENE, Chiba, Japan) and selected using hygromycin (Thermo Fisher Scientific, Waltham, MA, USA). Stable transfectants, designated JurkatΔαβCD8a-NFAT-RE-luc and JurkatΔαβCD8a-NFκB-RE-luc, respectively, were confirmed by reporter responsiveness to stimulation with 16 nM PMA (Sigma-Aldrich, St. Louis, MO, USA) and 14 μM Ionomycin (Adipogen Life Sciences, Fuellinsdorf, Switzerland) and used for CAR introduction. Male NSG (NOD. Cg-*Prkdc^scid^ Il2rg^tm1Wjl^*/SzJ) mice were purchased from Jackson Laboratories Japan (Kanagawa, Japan) and housed in the animal facility of Kanagawa Cancer Center Research Institute. Mice were purchased at 5 weeks of age and used for experiments before 8 weeks of age. FITC anti-CD3e (BioLegend Cat# 300405, RRID: AB_314059), PE/Cy7 anti-CD4 (BioLegend Cat# 317413, RRID: AB_571958), PerCP-Cy5.5 anti-CD4 (BioLegend Cat# 317427, RRID: AB_1186124), APC anti-CD8a (BioLegend Cat# 301014, RRID: AB_314132), PE-Cy7 anti-CD8a (BioLegend Cat# 301012, RRID: AB_314130) Pacific Blue anti-PD-1 (BioLegend Cat# 329915, RRID: AB_1877194), APC-Cy7 anti-Tim-3 (BioLegend Cat# 345025, RRID: AB_2565716), APC anti-LAG-3 (BioLegend Cat# 369211, RRID: AB_2728372), AlexaFluor 488 anti-CCR7 (BioLegend Cat# 353206, RRID: AB_10916389), APC CD62L (BioLegend Cat# 385105, RRID: AB_3097359), APC-Cy7 anti-CD45RA (BioLegend Cat# 304128, RRID: AB_10708880) and Pacific Blue anti-CD25 (BioLegend Cat# 356129, RRID: AB_2563589) monoclonal antibodies (mAbs) were obtained from BioLegend (San Diego, CA, USA). AlexaFluor594- or PE-labeled anti-G4S linker (Cell Signaling Technology; Cat# 38907, RRID: AB_3626304, Cat# 39614, RRID: AB_3626305) and anti-Whitlow/218 linker (Cell Signaling Technology; Cat# 62405, RRID: AB_3626306, Cat# 61465, RRID: AB_3626309) mAbs were purchased from Cell Signaling Technology (Danvers, MA, USA).

### 2.2 Construction of CAR-containing retrovirus vectors

Retroviral vectors containing CAR constructs for SKM-28z and SKM-BBz CARs, which have the (G4S)4 linker in scFv, have been described previously (4). The linker part of these SKM-(G4S)4-CAR vectors was modified to the Whitlow 218 linker sequence (GSTSGSGKPGSGEGSTKG) using Gibson assembly (New England Biolabs, Ipswich, MA). CD19-CARs were generated by replacing the SKM9-2 scFv sequence with the scFv sequence derived from anti-CD19 mAb FMC63 (7) linked by the Whitlow/218 linker sequence.

### 2.3 Expression of the CARs

**Figure 1:**
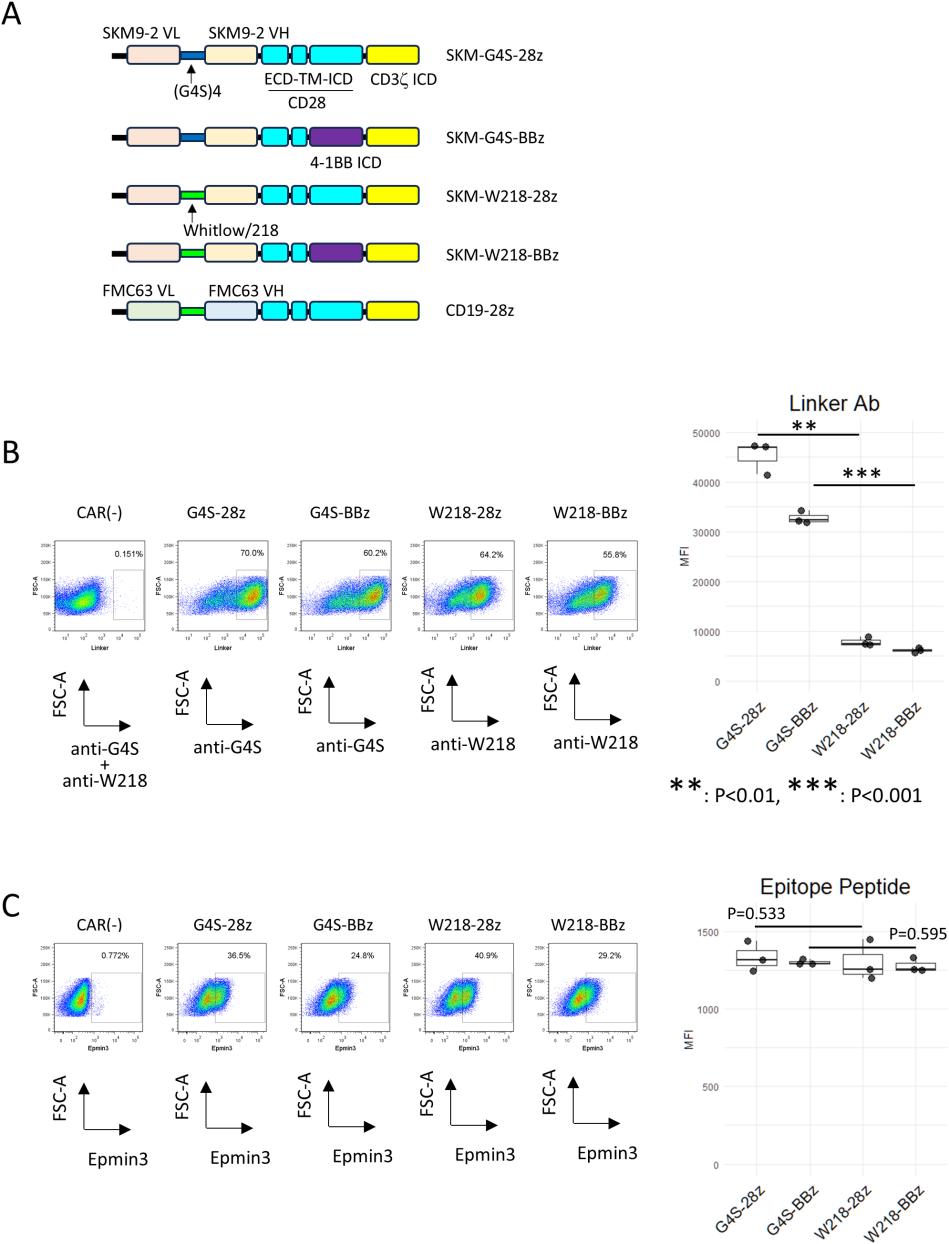
Expression of linker-modified CARs. **(A)** Constructs and names of the CARs used in this study. **(B)** Series of SKM-CARs with different scFv linkers were transduced into PBMC and analyzed by flow cytometry. CAR-T cells were stained with either PE-anti-(G4S)n linker mAb or PE-anti-Whitlow/218 linker mAb. The MFIs of each staining within the CAR+ gate were calculated from three biological replicates with different donor and shown in boxplots with individual data points (right panel). Significant differences were evaluated using a paired, two-tailed student’s T-test. **(C)** CAR-T cells were incubated with Epmin3 His x 6-tagged epitope peptide for SKM9-2 mAb, followed by staining with PE-anti-His tag antibody (left panels). The MFIs of each staining within the CAR+ gate were calculated from three independent experiments and shown in boxplots with individual data points (right panel). Significant differences were evaluated using a paired, two-tailed student’s T-test.

Human peripheral blood mononuclear cells (PBMCs) were collected by density gradient centrifugation (Lymphoprep; Alere Technologies AS, Oslo, Norway) from the peripheral blood of three independent healthy volunteers who provided written informed consent. The study was conducted in accordance with the tenets of the Declaration of Helsinki, and the protocol was approved by the Institutional Review Board of Kanagawa Cancer Center (study number: 28-KEN-29). PBMCs (1×10^6^ cells/mL) were stimulated using plate coated with 5ug/ml anti-CD3 mAb (clone UCHT-1; BD Biosciences, San Jose, CA, USA) and 10 ng/mL human IL-2 (Peprotech, Rocky Hill, NJ, USA) for 2 days in AIM-V medium (Thermo Fisher Scientific) supplemented with 10% human AB serum (MP Biomedicals, Irvine, CA, USA). A retroviral vector containing CAR cDNA was transfected into Phoenix-Ampho packaging cells using X-fect reagent (Takara Bio, Shiga, Japan), according to the manufacturer’s instructions, to obtain the virus-containing supernatant. The virus-containing supernatant was added to an untreated 24-well plate coated with retronectin (Takara Bio) and centrifuged at 2000 ×g for 2 hours at 32°C to facilitate the binding of virus particles to retronectin. Activated PBMCs (2.5×10^5^ cells/well) were then added to the virus-coated plates for infection. CAR-T cells expressing SKM-28z CAR with the (G4S)_4_ linker, SKM-BBz CAR with the (G4S)_4_ linker, SKM-28z CAR with the Whitlow/218 linker, and SKM-BBz CAR with the Whilow/218 linker were designated SKM-G4S-28z, SKM-G4S-BBz, SKM-W218-28z, and SKM-W218-BBz, respectively (**Figure 1A**). Twenty-four hours later, secondary infection was performed, and cells were cultured with medium containing 10ng/ml of human IL-2 and passaged every 3 to 4 days at 5x10^5^ cells/ml.

CAR antigen specificity was assessed by incubation with the SKM-Epmin3 peptide, a His-tagged epitope of HEG1 recognized by the SKM9-2 mAb (9), followed by staining with a PE-labeled anti-His tag Ab (BioLegend Cat# 362603, RRID: AB_2563634). CAR expression on PBMCs and the surface phenotypes of CAR+ cells were determined by multicolor flow cytometry using PE anti-G4S or anti-Whitlow/218 linker mAb, PerCP-Cy5.5 anti-CD4 mAb, FITC or PE-Cy7 anti-CD8a mAb, APC-Cy7 anti-Tim-3 mAb, APC anti-LAG3 mAb, Pacific Blue anti-PD-1 mAb, AlexaFluor 488 anti-CCR7 mAb, APC anti-CD62L mAb, APC-Cy7 anti-CD45RA mAb and Pacific Blue anti-CD25 mAb. For the *in vitro* cytotoxicity assay, CAR-positive cells were sorted using a FACS Aria II cell sorter (BD Biosciences) after staining with PE anti-linker mAbs. For CAR expression in Jurkat reporter cell lines, the reporter cells (1 × 10^5^ cells) were single-infected with a retronectin-bound CAR-encoding retrovirus in a 24-well plate.

To image CAR distribution on the cell surface, CAR-T cells were stained with Alexa Fluor 594-labeled rabbit mAbs against the corresponding linkers in scFv (i.e. anti-G4S for G4S-CARs and anti-Whitlow/218 linker for W218-CARs). Cells were fixed with 4% formaldehyde and embedded in VECTASHIELD Antifade mounting medium (Vector Laboratories, Newark, CA, USA) in a glass-bottom dish (MatTek Life Sciences, Ashland, MA, USA). Confocal images were captured using a Zeiss LSM 710 (Carl Zeiss AG, Oberkochen, Germany) equipped with a 63x/1.4 NA PlanApo objective in 1.0 μm sections from cell top to bottom. For quantitative analysis, regions of interest (ROI) were defined on the cell surface area of the equatorial section image, and mean fluorescence intensities (MFI) and standard deviations (SDs) were obtained using Fiji software (RRID: SCR_002285) (10). To quantify the relative variability of CAR expression, the coefficient of variation (CV=SD/MFI) was calculated for 12 cells of each CAR (11).

### 2.4 *In vitro* cytotoxicity assay and measurement of cytokine levels in culture supernatants

ACC-MESO-4 cells (5 × 10^3^ cells) were mixed with sorted CAR-T cells (5 × 10^3^ to 2.5 × 10^4^ cells; at E:T ratios from 1:1 to 5:1) in a 96-well V-bottom culture plate and cultured for 18 h. The amount of LDH in the culture supernatant was measured using the cytotoxic LDH assay kit-WST (Dojindo, Kumamoto, Japan). Specific LDH release was calculated by comparison with the maximum LDH release after lysis with 0.5% Triton X-100. The *in vitro* cytotoxicity kinetics were measured using an xCELLigence real-time cell analyzer (Agilent, Santa Clara, CA, USA). ACC-MESO-4 cells (2 × 10^4^ cells) were seeded onto impedance plates and cultured overnight. After 22 h, the CAR-T cells (2 × 10^4^ cells/well) were added to each well. The impedance of each culture well was measured continuously for 28 h. The cytokine levels in the culture supernatant of the LDH release assay were evaluated using a bead-based multiplex assay. In this assay, cytokines were measured in 50 μL aliquots of 4-fold diluted supernatant of E:T=5:1 cultures using the Bio-Plex 200 system (Bio-Rad Laboratories, Hercules, CA). The Bio-Plex Pro Human Cytokine Th1/Th2 Assay (Bio-Rad Laboratories) was used to measure the following 9 cytokines: IL-2, IL-4, IL-5, IL-10, IL-12 (p70), IL-13, GM-CSF, IFNγ, and TNF-α. IL-2 measurement was not evaluated because the culture media contained 10 ng/mL recombinant human IL-2, making it difficult to measure the IL-2 secreted by CAR-T cells. In some experiments, remaining cells after killing assay at E:T = 5:1 condition were recovered and stained for FACS analysis.

### 2.5 Reporter assay

To quantify NFAT or NFκB reporter activity, CAR-expressing JurkatΔαβCD8a-NFAT-RE-luc cells or JurkatΔαβCD8a-NFκB-RE-luc cells (2×10^5^ cells) were seeded in 96-well white plates. BrightGlo luciferase assay substrate (Promega) was added to the cultures, and luminescence was measured using an Arvo plate reader (PerkinElmer, Waltham, MA, USA). For antigen stimulation, ACC-MESO-4 cells (1×10^4^ cells) were seeded in 96-well white plates one day before the assay. CAR-T cells (1×10^4^ cells) were added and co-cultured for 5 h before luciferase activity measurement.

### 2.6 RNA sequencing analysis

CAR-infected PBMCs were stained with an anti-mouse IgG polyclonal antibody, and CAR-positive cells were sorted on day 9 after infection using a FACSAria II cell sorter. After two days of culture, total RNA was purified from the sorted cells using an RNeasy Mini RNA Extraction Kit (Qiagen, Germantown, MD, USA). Three biological replicates were used for this preparation. The cDNA library was generated using the SMART-Seq v4 Ultra Low Input RNA kit (TaKaRa Bio), sequenced using a NovaSeq 6000 sequencer (Illumina, San Diego, CA), and mapped to GRCh38 [GENCODE v42 (RRID: SCR_014966)] using KI Biobank - STAR (2.7.10b) (RRID: SCR_005923). Principal component analysis (PCA) was performed using the DESeq2 software (RRID: SCR_015687) (12). Based on the transcriptome data, differentially expressed genes (DEGs) were identified using DESeq2 with the criteria of q<0.05, p<0.05, and |log2FC|>1.

### 2.7 *In vivo* transfer experiment

In an ectopic mouse model, 25 male NSG mice were subcutaneously injected with ACC-MESO-4 cells (1x10^6^ cells), 7 days later (day 0), mice were randomly divided into 4 groups and each group received intravenous injection of G4S-28z or W218-28z CAR-T cells (1x10^6^ cells) or left untreated (control) (n=6~7/group). Tumor sizes were measured using calipers, and tumor volumes were calculated using the following formula: (long axis) x (short axis)^2^ x 1/2. Exact Wilcoxon-Mann-Whitney test was used to statistically evaluate tumor volumes at the end of observation (day 70). P values < 0.05 were considered statistically significant. During the experiments, no mice reached the prespecified exclusion criteria of tumor volume greater than 1500mm^3^ or 10% weight loss.

## 3 Results

### 3.1 Comparable surface expression of linker-modified SKM-CARs

PBMC transduced with SKM-G4S-28z or SKM-G4S-BBz CAR were positively stained with an antibody against the G4S linker. Conversely, cells transduced with SKM-W218-28z and SKM-W218-BBz CAR were positively stained with an antibody against the Whitlow/218 linker, indicating the successful construction of the retroviral vectors (**Figure 1B**). We observed that the staining intensities of W218 CARs were weaker than those of G4S CARs. This may be because of the different affinities of each mAb for the corresponding epitopes. To directly compare the expression levels of each CAR type and confirm the antigen specificities of the modified CARs, CAR-T cells were incubated with a (His)6-tagged epitope peptide for SKM9-2 mAb. As shown in **Figure 1C**, CAR-T cells with different linkers showed no significant differences in epitope peptide staining, indicating that the modified CARs retained their original antigen specificity and had comparable expression levels. During CAR-T cell production, all CAR-T cells proliferated equally to CAR (–) cells, while W218 CAR-T cells tended to show lower viability compared to G4S CAR-T cells. (**Supplementary Figure S1**).

### 3.2 Linker-modification of SKM-CARs did not alter cell surface CAR aggregation

**Figure 2:**
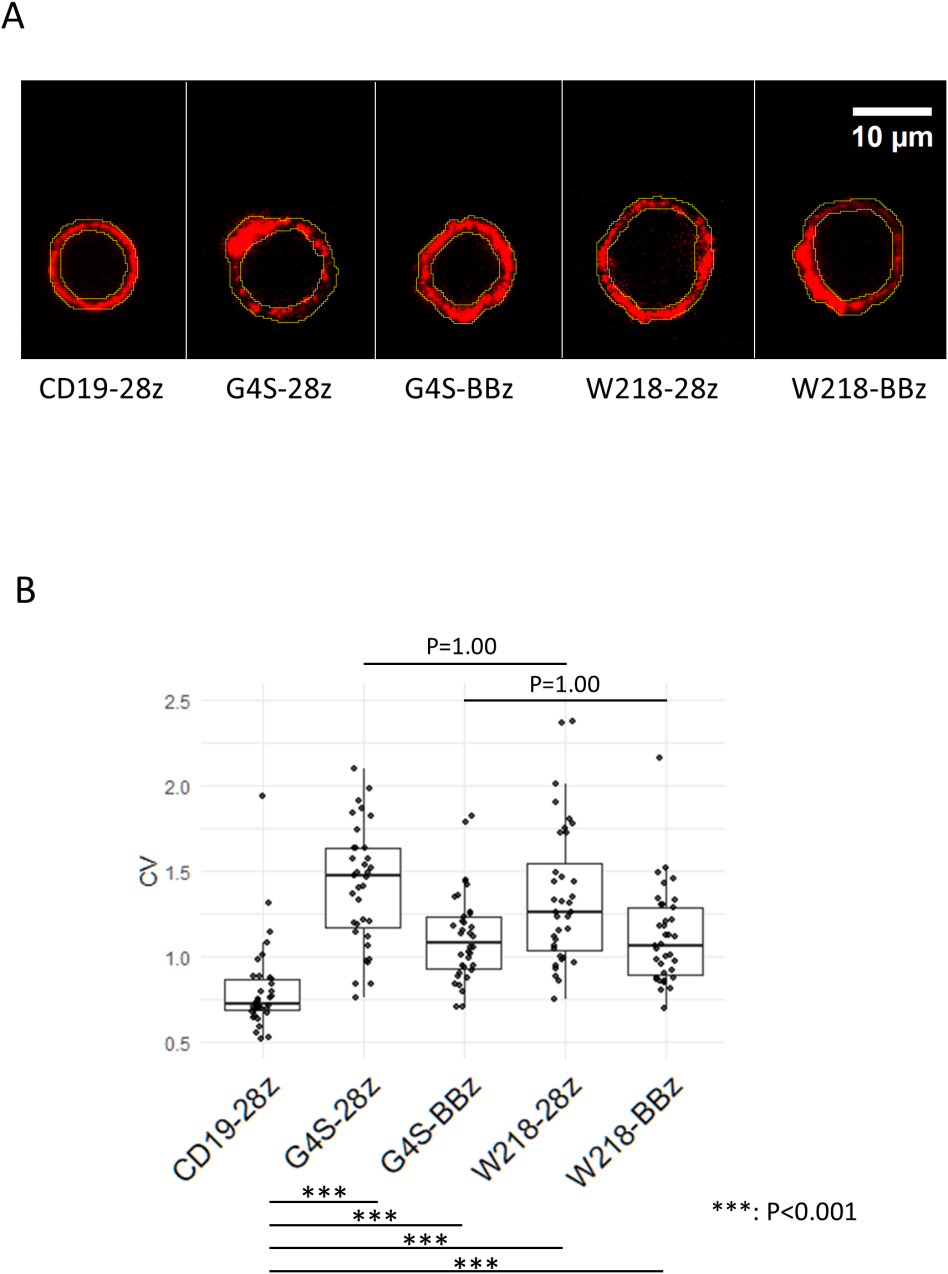
Aggregation of linker-modified CARs on the cell surface. **(A)** Series of SKM-CARs with the different scFv linkers were transduced into PBMCs and analyzed by confocal microscopy. Representative images of the cell surface of each CAR are shown. Regions of interest (ROI) were defined on the cell surface in the images at the equatorial section as shown in yellow. **(B)** MFIs of the ROI were obtained for 12 cells per CAR, and the coefficient of variation (CV = SD/MFI) for each CAR was calculated and shown in the Boxplots with individual data points. Significant differences in CVs were evaluated by non-paired, two-tailed Student’s T-test.

In our previous study, SKM-G4S-CARs, independent of intracellular co-stimulatory domains, showed a tendency to aggregate on the cell surface compared to CD19-CAR with the Whitlow/218 linker (4). To determine whether the introduction of the Whitlow/218 linker into SKM-CARs reduced their tendency to aggregate on the cell surface, the cell surface distribution of each CAR was observed using confocal microscopy (**Figure 2A**). Modification of the linker sequence did not affect the CV values of cell surface CAR staining. However, all SKM-CARs were significantly more aggregated than CD19-CAR with the Whitlow/218 linker (**Figure 2B**).

### 3.3 Linker-modification did not affect cell surface phenotypes of SKM-CAR T cells

**Figure 3:**
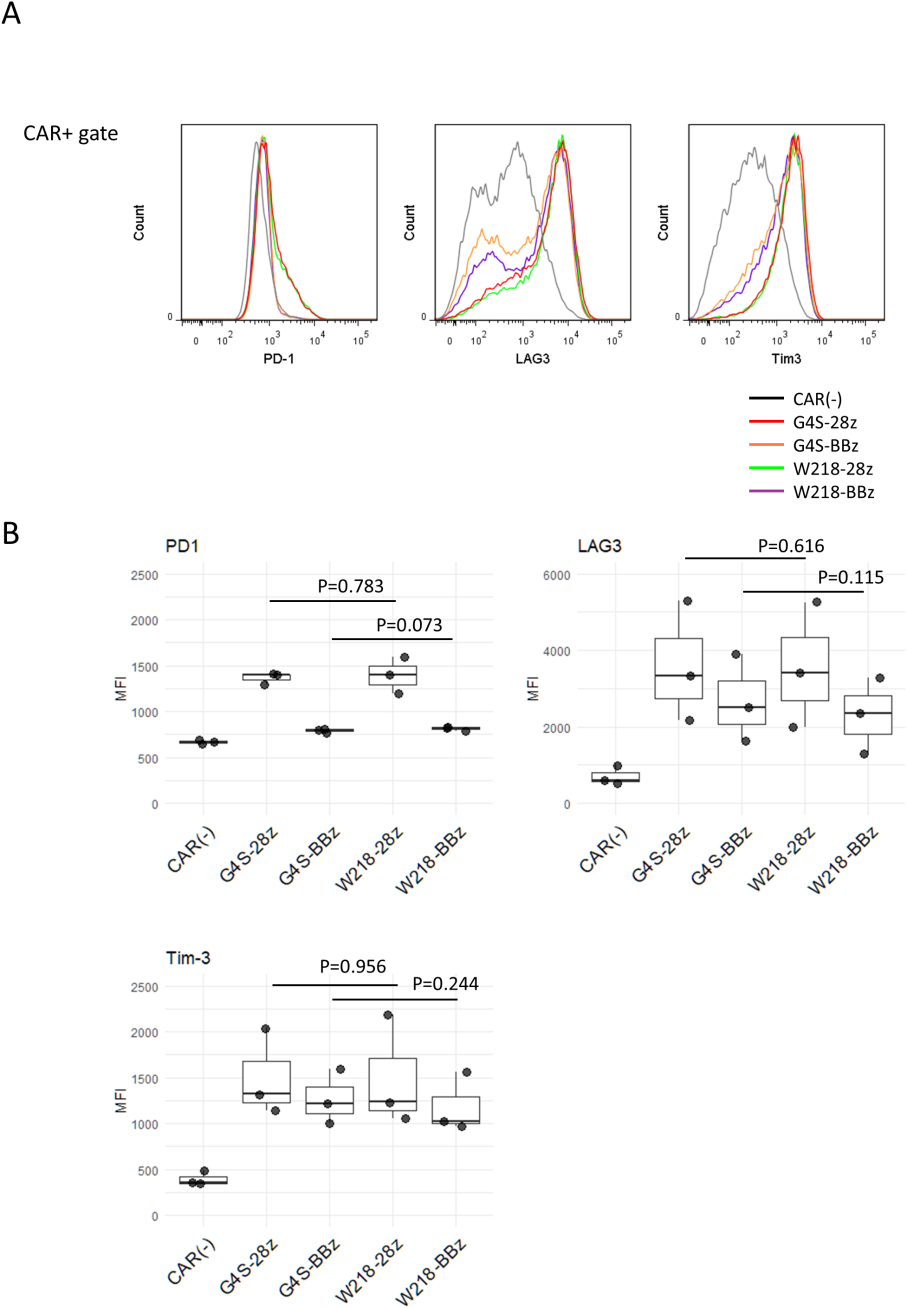
Surface expression of exhaustion markers on CAR-T cells. Eleven days after retroviral infection, CAR-T cells were stained with anti-PD1, anti-LAG3, and anti-Tim-3 mAbs together with mAbs against their respective linkers conjugated with separate fluorochromes. **(A)** Histograms of PD-1, LAG3, and Tim-3 expression within the CAR+ gate. Representative plots of three independent experiments are shown. The CAR+ gate was omitted from the CAR (–) cells. **(B)** MFIs for PD-1, LAG3, and Tim-3 staining in CAR+ cells were calculated from samples from three independent experiments. Boxplots with individual data points are presented. Significant differences were evaluated using a non-paired, two-tailed Student’s t-test.

In our previous study, SKM-G4S-28z CAR-T cells expressed higher levels of T cell exhaustion markers, Tim-3, LAG3, and PD-1, than CD19-CAR-T cells due to spontaneous signaling independent of antigen ligation (tonic signal). SKM-G4S-BBz CAR-T cells also generated a tonic signal, but with a different quality caused by the 4-1BB co-stimulatory domain and did not upregulate exhaustion markers. To investigate the influence of linker sequences on tonic signal-induced surface phenotypes, the expression of Tim-3, LAG3, and PD-1 on SKM-W218 CAR-T cells was compared with that on SKM-G4S CAR-T cells. Consistent with the above observation that there was no difference in the cell surface aggregation of G4S and W218 CARs, SKM-W218-28z CAR-T cells showed similar upregulation of these T cell exhaustion markers as SKM-G4S-28z CAR-T cells (**Figure 3**). Expression of exhaustion markers were almost the same between CD8^+^ and CD4^+^ CAR-T cells except for LAG3, which is expressed much higher in the CD8^+^ CAR-T cells (**Supplementary Figure S2**). The expression of exhaustion markers were up-regulated at day 11 compared to day 4 from retrovirus infection, suggesting these are induced by persistent tonic signaling from SKM-CARs (**Figure S3**). To further address changes in the phenotypes of CAR-T cells with different CAR constructs, T cells differentiation markers CCR7, CD62L and CD45RA, as well as activation marker CD25 were stained. All CAR-T cells showed decrease in the CD62L and CD45RA compared to CAR (–) control, suggesting differentiation from naïve stage. Interestingly the two SKM-BBz CAR-T cells showed remarkable upregulation of CCR7 compared to the two SKM-28z CAR-T cells, suggesting differentiational arrest before effector memory/terminal exhausted stage (**Supplementary Figure S4**). While these phenotypes were consistent with our previous report, again, there is no noticeable difference between G4S CAR-T cells and W218 CAR-T cells. Expression of CD25 were upregulated in all SKM-CAR-T cells compared to CAR (–) control, suggesting presence of the tonic signal.

### 3.4 Effect of the linker sequences on the tonic signal from SKM-CARs

**Figure 4:**
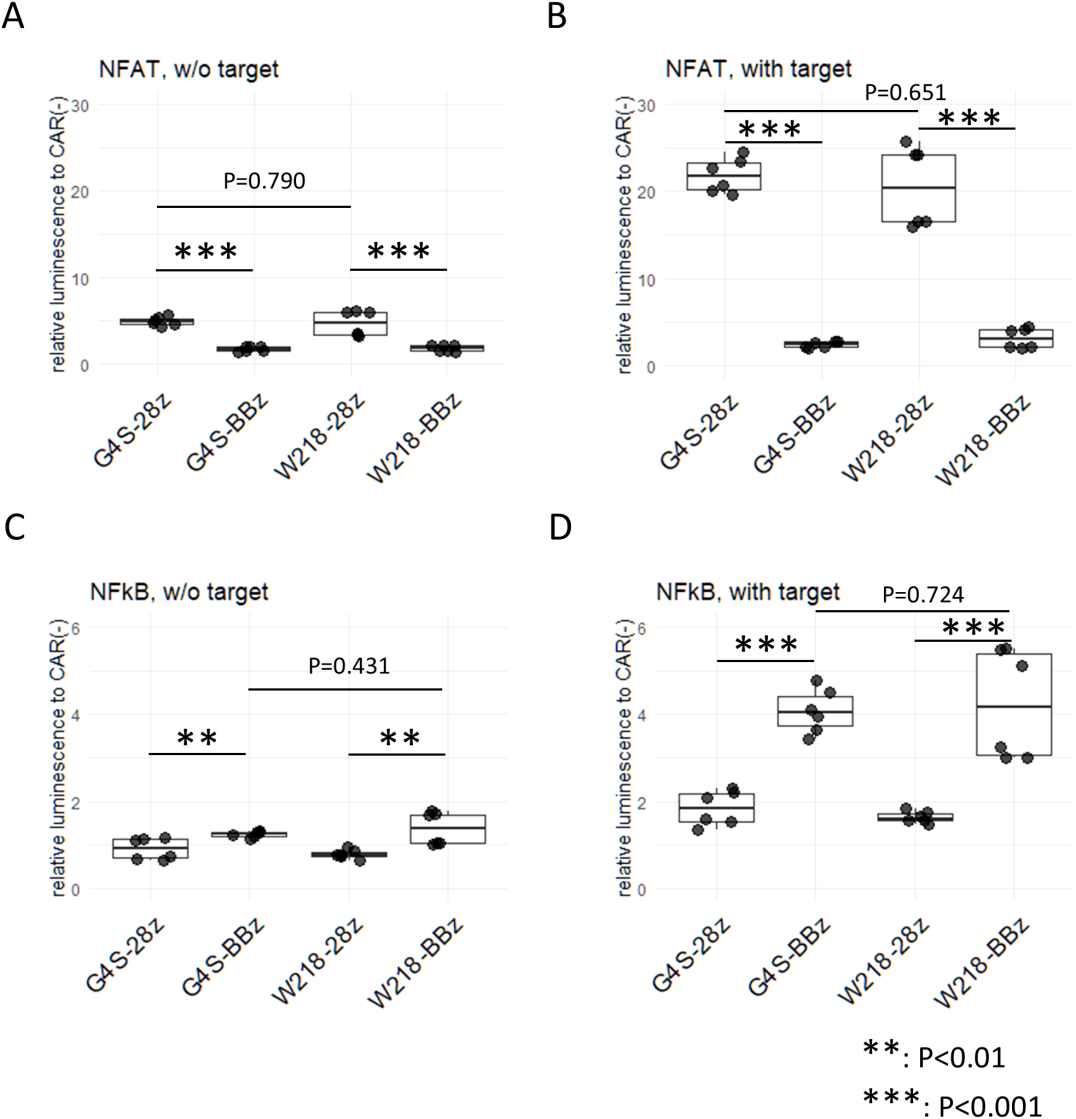
Reporter assay for NFAT and NFκB in CAR-expressing Jurkat cell lines with and without stimulation. JurkatΔαβCD8a-NFAT-RE-luc **(A, B)** and JurkatΔαβCD8a-NFκB-RE-luc **(C, D)** cells were transduced with each CAR, and luciferase activity was measured with **(B, D)** or without **(A, C)** coculture with ACC-MESO4 cells for 5 hours. Luminescence measurements were divided by those without CAR expression to obtain the relative luminescence. Boxplots with individual data points of two triplicate experiments were calculated. Significant differences were evaluated using a non-paired, two-tailed Student’s t-test.

To investigate the downstream signaling pathways induced by tonic signals in each CAR construct, CAR molecules were expressed on TCR-deficient Jurkat cells transduced with the NFAT reporter gene, called JurkatΔαβCD8a-NFAT-RE-luc cells. Consistent with our previous results, strong NFAT activation was observed in the JurkatΔαβCD8a-NFAT-RE-luc cells expressing SKM-28z CARs, but almost no NFAT activity was detected in those expressing SKM-BBz CARs (**Figure 4A**). Notably, cells expressing either SKM-G4S-28z or SKM-W218-28z showed NFAT activation at the same level, suggesting that there was no change in the magnitude of tonic signals in SKM-28z CARs with different linkers (**Figure 4A**). Similarly, the level of NFAT reporter activity after 5 h of antigen stimulation was not different between the SKM-G4S-28z and SKM-W218-28z CARs (**Figure 4B**).

To investigate another downstream signaling pathway, NFκB, in each CAR construct, CARs were expressed on TCR-deficient Jurkat cells with the NFκB reporter gene, i.e., JurkatΔαβCD8a-NFκB-RE-luc cells. NFκB reporter activity was significantly upregulated in the SKM-CAR-expressing cells without stimulation (**Figure 4C**) and was increased after antigen stimulation: SKM-BBz CARs showed stronger activities than SKM-28z CARs after antigen stimulation (**Figure 4D**). Notably, the levels of NFκB signals between SKM-BBz CARs with the different linkers as well as between SKM-28z CARs with the different linkers were not significantly different (**Figure 4C, D**).

### 3.5 Enhancement of the *in vitro* killing activity of CAR-T cells expressing SKM-28z CAR with the Whitlow/218 linker

**Figure 5:**
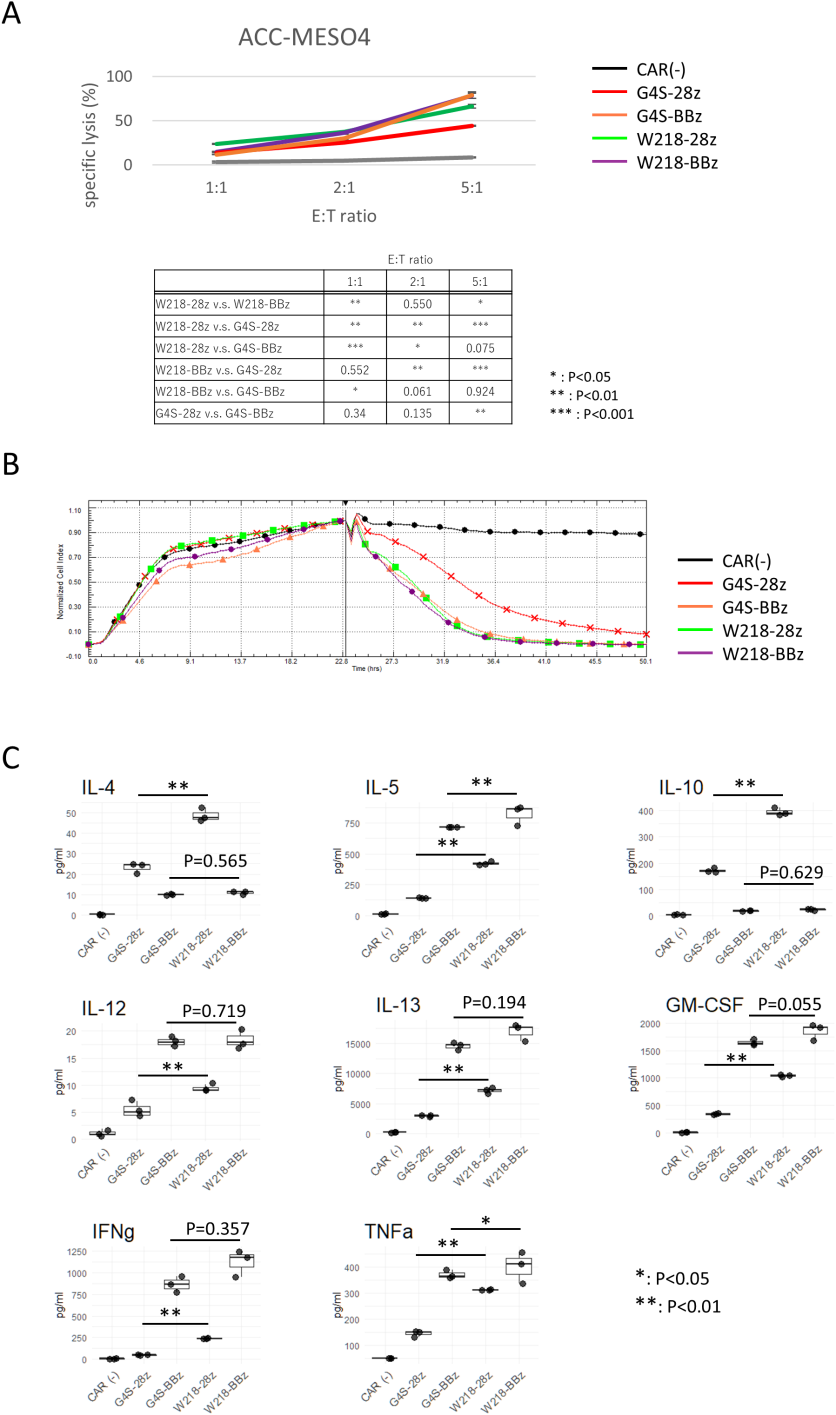
*In vitro* killing activity and cytokine production of CAR-T cells. **(A)** CAR-T cells were co-cultured with ACC-MESO4 cells at the E:T ratio as shown. After 18 h, the amount of LDH in the culture supernatant was measured, and the specific lysis % was calculated as described in the Materials and Methods. The averages and standard deviations of three biological replicates are shown. The values are representative of three independent experiments showing similar results. Significant differences were evaluated using a non-paired, two-tailed Student’s T-test. **(B)** ACC-MESO4 cells were cultured in the impedance plate with continuous measurement of the impedance. After 22 hours, CAR-T cells were added to the wells, and the impedance was monitored for another 28 hours. A representative plot of three replicates with different PBMC donors is shown. **(C)** Cytokine production profile of CAR-T cells during the target killing reaction at E:T=5:1 was measured using culture supernatants collected at the end of LDH assay. Boxplots with individual data points of three biological replicates are shown. Representative of three independent experiments showing similar results. Significant differences were evaluated using a non-paired, two-tailed Student’s T-test.

To compare the *in vitro* killing activity of CAR-T cells expressing each CAR construct, CAR-T cells were cocultured with ACC-MESO4 cells expressing the SKM9-2 antigen. Consistent with our previous results obtained using the G4S linker (4), SKM-G4S-28z CAR-T cells tended to have a lower ability to kill ACC-MESO4 cells than SKM-G4S-BBz CAR-T cells. In contrast, in the case of the Whitlow/218 linker, both SKM-W218-28z and SKM-W218-BBz CAR-T cells showed good killing abilities, comparable to those of SKM-G4S-BBz CAR-T cells (**Figure 5A**). Because the LDH assay can only detect endpoint differences, the killing kinetics were measured using a real-time cell analyzer that detects plate impedance. Consistent with our previous report, SKM-G4S-28z CAR-T cells (red) exhibited slower kinetics than SKM-G4S-BBz CAR-T cells (orange). Notably, SKM-W218-28z CAR-T cells (green) exhibited similar kinetics to SKM-G4S-BBz (orange) and SKM-W218-BBz CAR-T cells (purple). This result demonstrated that replacing the G4S linker with the W218 linker improved the killing activity of SKM-28z CAR-T cells, whereas there were no differences in CAR aggregation, cell surface phenotypes, or tonic signal intensity between them. Following the differences in *in vitro* killing ability despite the lack of differences in surface antigens, the phenotype of CAR-T cells after killing target cells was examined. As expected, the surface expression of exhaustion markers (PD-1, LAG3 and Tim-3) was upregulated after target cell killing. However, there was no difference between G4S CAR T cells and W218 CAR T cells in the degree of exhaustion marker upregulation (**Supplementary Figure S5**).

### 3.6 Differences in cytokine secretion by activated CAR-T cells with the different linkers

To further investigate the functional differences between SKM-CAR-T cells with the G4S and Whitlow/218 linkers, cytokine secretion from activated CAR-T cells was measured (**Figure 4C**). Culture supernatants from CAR-T cells co-cultured with ACC-MESO4 cells were applied to a bead-based multiplex assay to quantify T cell cytokines: IL-4, IL-5, IL-10, IL-12 (p70), IL-13, GM-CSF, IFNγ, and TNF-α. There were considerable differences in the secretion of each cytokine between SKM-G4S-28z and SKM-G4S-BBz CAR-T cells; SKM-G4S-28z CAR-T cells secreted more IL-4 and IL-10 than SKM-G4S-BBz CAR-T cells, while SKM-G4S-BBz CAR-T cells secreted more IL-5, IL-12, IL-13, GM-CSF, IFNγ, and TNF-α. A comparison between SKM-W218-28z and SKM-W218-BBz CAR-T cells revealed the same trend. Notably, when comparing SKM-G4S-28z and SKM-W218-28z CAR-T cells, SKM-W218-28z CAR-T cells secreted significantly higher levels of all cytokines tested than SKM-G4S-28z CAR-T cells did.

### 3.7 Gene expression of SKM-CAR-T cells with different linker sequences

**Figure 6:**
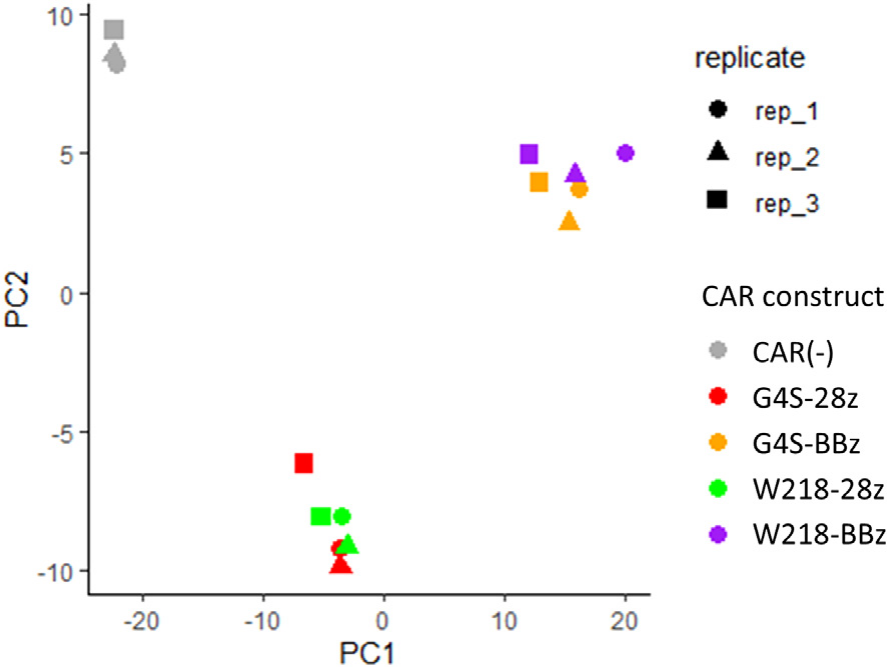
PCA plot of transcriptome data from CAR-T cells in three independent experiments. The CAR constructs and replicate numbers are classified by color and shape, respectively, as indicated in the figure.

To investigate the molecular changes caused by switching the linker sequence from G4S to Whitlow/218, the gene expression of each CAR-T cell in the absence of antigen stimulation was compared using RNA sequencing. CAR-T cells expressing SKM-G4S-28z CAR or SKM-G4S-BBz CAR and T cells without CAR expression exhibited different gene expression patterns in the PCA plot (**Figure 6**). Interestingly, SKM-W218-28z and SKM-W218-BBz CAR-T cells showed gene expression patterns similar to those of their respective counterparts with the G4S linker. When DEGs were identified using the criteria |log2FC>1, p<0.05 and q < 0.05, 16 DEGs were observed between SKM-W218-28z and SKM-G4S-28z CAR-T cells (**Table 1**), whereas there were only four DEGs between SKM-W218-BBz and SKM-G4S-BBz CAR-T cells (**Table 2**). These results suggest that no significant differences were observed in most genes owing to the substitution of linker sequences at steady state without stimulation.

**Table 1:** Differentially expressed genes between SKM-W218-28z CAR-T cells and SKM-G4S-28z CAR-T cells.

Gene Name	log2 FC	p value	q value
IL5	-2.959239253	3.79E-09	5.87E-05
FAM131C	-1.985466495	7.52E-09	5.87E-05
KCNQ5	1.637715855	6.78E-07	0.00211686
TXNRD3	3.720031814	1.53E-06	0.003983062
BBS9	1.246913686	2.97E-06	0.005785747
ENSG00000284773	-4.715252467	3.96E-06	0.006873586
SPAG6	-3.795989802	4.90E-06	0.007646405
ZNF182	1.603707142	6.47E-06	0.009173211
TSPOAP1	-3.224610524	1.16E-05	0.013897518
CCT6B	1.778416697	1.84E-05	0.017916081
PRR5-ARHGAP8	3.586538135	2.95E-05	0.027109059
LINC02693	-1.896010594	3.59E-05	0.027879431
LTB	-1.004632924	4.18E-05	0.029566339
CXCL8	-1.486087372	4.58E-05	0.029784871
DPEP2	1.026395137	9.67E-05	0.044360133
HHLA2	-1.927191585	0.000113567	0.049228125

Differentially expressed genes (DEGs) between SKM-W218-28z and SKM-G4S-28z CAR-T cells (N = 3, |log2FC| > 1, q < 0.05, p < 0.05) are listed. FC=SKM-G4S-28z / SKM-W218-28z.

**Table 2:** Differentially expressed genes between SKM-W218-BBz CAR-T cells and SKM-G4S-BBz CAR-T cells.

	log2 FC	p value	q value
MCM9	-1.629104643	3.96E-07	0.003087
PLCL1	3.687382835	1.43E-05	0.037286
SCRN1	-4.008345609	2.49E-05	0.043142
GRAMD2B	-1.179408795	6.74E-06	0.021031

Differentially expressed genes (DEGs) between SKM-W218-BBz and SKM-G4S-BBz CAR-T cells (N = 3, |log2FC| > 1, q < 0.05, p < 0.05) are listed. FC=SKM-G4S-28z / SKM-W218-28z.

### 3.8 Effect of linker substitution in tumor control ability *in vivo*

**Figure 7:**
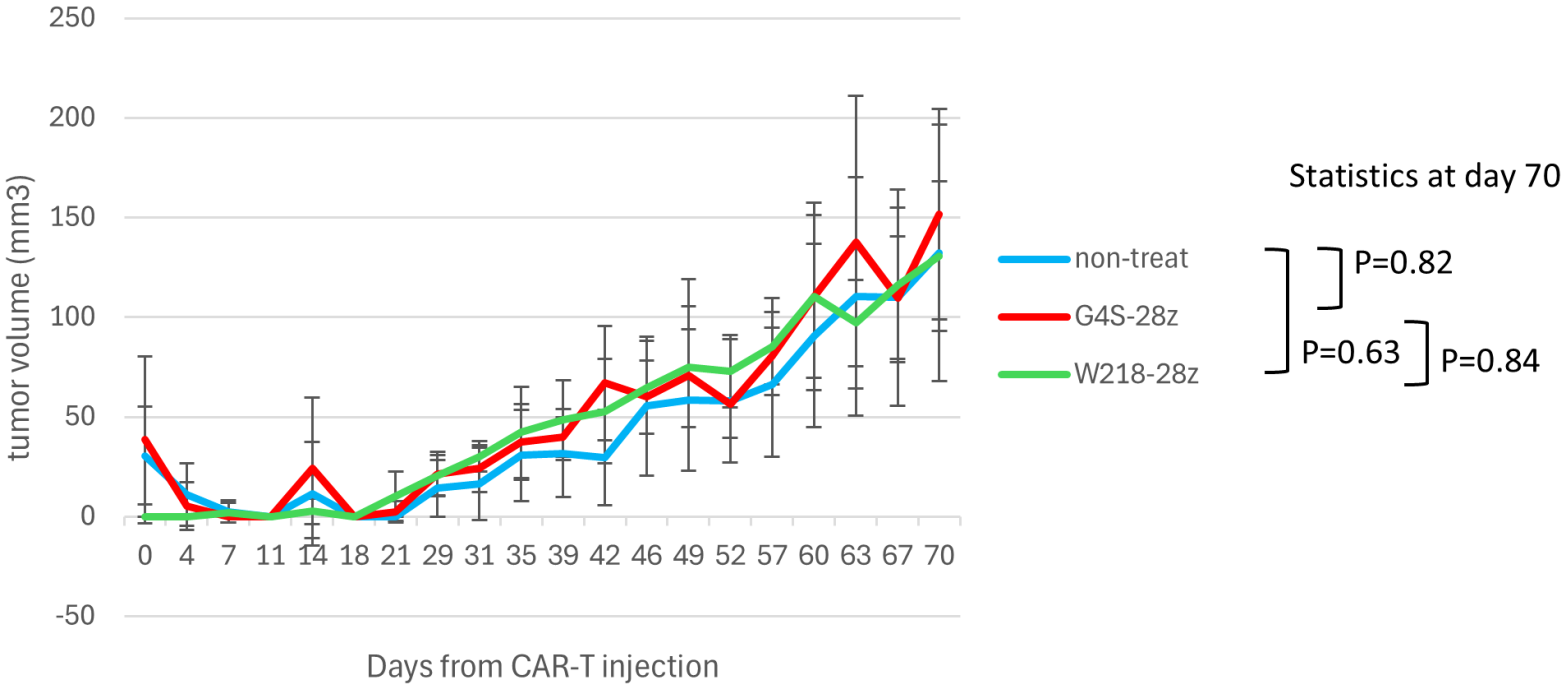
*In vivo* tumor control of linker-substituted CAR-T cells. NSG mice were injected subcutaneously with ACC-MESO-4 cells (1x10^6^ cells), followed by intravenous injection of the sorted CAR-T cells (1x10^6^ cells) 7 days later (day 0). Tumor sizes were measured using calipers until day 70, and tumor volumes were calculated using the following formula: (long axis) x (short axis)^2^ x 1/2. The mean and standard error of tumor volumes after treatment with G4S-28z and W218-28z CAR-T cells and those of untreated controls (n=6~7, each group) are shown. The exact Wilcoxon-Mann-Whitney test was employed to statistically evaluate tumor volumes at day 70.

We investigated the ability of SKM-G4S-28z and SKM-W218-28z CAR-T to control tumor growth *in vivo* using a human tumor cell line xenograft model. The ectopic xenograft model was established by subcutaneous injection of the malignant mesothelioma cell line ACC-MESO-4 into immunodeficient NSG mice. Unexpectedly, despite the improved *in vitro* killing activity (**Figure 5A, B**), intravenous administration of SKM-W218-28z CAR-T did not delay tumor growth compared to SKM-G4S-28z CAR-T treated or untreated mice, as shown in **Figure 7**. At the endpoint of the mouse experiment, we isolated cells from tumors or spleens and examined CAR expression in the isolated cells by flow cytometry using mAbs against the G4S or W218 linker. However, no CAR-T cells were detected in the tumors or spleens of SKM-W218-28z or SKM-G4S-28z CAR-T treated mice (data not shown).

## 4 Discussion

In this study, we investigated the influence of substitution of linker sequences from G4S to Whitlow/218 in the self-activating CAR construct. Substitution did not induce changes in surface aggregation of CAR, surface phenotype of CAR T cells, or intensity of downstream NFAT and NFκB signaling. However, SKM-28z CAR-T cells with the Whitlow/218 linker showed improved *in vitro* killing activity against target cells as well as increased effector cytokine secretion upon target engagement.

Constitutive CAR signaling induces the terminal differentiation and dysfunction of CAR-T cells (1). Recently, we reported that CAR-T cells expressing constitutively active SKM-28z CAR tended to undergo terminal exhaustion in the absence of exogenous stimulation. Substitution of the CD28 co-stimulatory domain with 4-1BB has been shown to be effective in reducing spontaneous differentiation to the exhausted stage (4). Temporary cessation of continuous CAR signaling by the downregulation of CAR expression or pharmacological inhibitors has also been reported to reverse the spontaneous terminal differentiation of CAR-T cells (13). Since the constitutive activation of CAR is caused by the spontaneous aggregation of CAR molecules on CAR-T cells (1), the elimination of self-aggregation without altering antigen specificity would be an attractive means to produce more efficient CAR-T cells. Self-aggregation of CAR molecules depends on the extracellular domain of CAR, specifically the frame region of the scFv portion (1). CTL019 (tisagenlecleucel) CAR is one of the CARs with a very low tonic signal. Whereas most CARs use the G4S linker in their scFv portion, CTL019 uses a Whitlow/218 linker (6). The Whitlow/218 linker showed higher resistance to proteolysis and reduced aggregation (14) than the Whitlow/212 linker, which was selected based on criteria related to protein folding, stability, and antigen-binding characteristics (15). Although the Whitlow/218 linker has not been directly compared with the G4S linker in self-activating CARs with tonic signals, we speculate that the Whitlow/218 linker may reduce aggregation and tonic signaling. Therefore, we modified the self-activating SKM-CARs by replacing the G4S linker with the Whitlow/218 linker.

SKM-CARs with the Whitlow/218 linker, SKM-W218-28z and SKM-W218-BBz CARs, were expressed at levels almost comparable to the original SKM-G4S-28z and SKM-G4S-BBz CARs and retained their original antigen specificity, suggesting that the Whitlow/218 linker can function for scFv generation by SKM9-2 mAb. When the self-aggregation tendency of each CAR was evaluated by confocal microscopy, CD19 CAR showed a uniform distribution on the surface of CAR-T cells, whereas SKM-G4S-28z and SKM-G4S-BBz CARs formed aggregates on CAR-T cells, which resulted in significantly higher coefficient of variation (CV) values of fluorescence intensity on the cell membrane for SKM-G4S CARs. Contrary to our expectations, SKM-CARs with the Whitlow/218 linker, SKM-W218-28z and SKM-W218-BBz CARs, did not show a significant reduction in CV values compared with their respective G4S counterparts. In support of these observations, the surface phenotypes of SKM-W218 CAR-T cells did not differ from those of SKM-G4S CAR-T cells. In addition, the NFAT and NFκB reporter assays also indicated that there were no qualitative or quantitative changes in the downstream signaling of SKM-G4S and SKM-W218 CARs. These results suggested that the Whitlow/218 linker did not reduce cell surface aggregation or tonic signaling in SKM-CARs.

However, the substitution of the linker sequence from G4S to Whitlow/218 improved the *in vitro* killing activity of SKM-28z CAR-T cells. In addition, when the cytokine secretion profiles were compared, SKM-W218-28z CAR-T cells secreted significantly higher levels of all cytokines examined than SKM-G4S-28z CAR-T cells, although the levels of cytokines, except for IL-4 and IL-10, secreted from SKM-W218-28z CAR-T cells were not as high as those secreted from SKM-G4S-BBz or SKM-W218-BBz CAR-T cells. These results suggest that, although there is little difference in steady-state CAR-T cells with different linker sequences, differences in killing activity and cytokine production become significant upon antigen stimulation. Notably, among these cytokines, two effector cytokines, IFNγ and TNF-α, may be directly associated with a greater ability to kill target cells in SKM-W218-28z CAR-T cells. In addition, the less functional SKM-G4S-28z CAR-T cells secreted more IL-4 and IL-10 than the functional SKM-G4S-BBz and SKM-W218-BBz CAR-T cells. IL-10 is an immunoregulatory cytokine that may contribute to the functional limitations of SKM-G4S-28z CAR-T cells. However, SKM-W218-28z CAR-T cells secreted more IL-4 and IL-10 than SKM-G4S-28z CAR-T cells, raising the question of the role of these cytokines in CAR-T cell function.

When the transcriptome profiles of CAR-T cells were compared by PCA plot, they were almost indistinguishable between SKM-G4S and SKM-W218 CARs at steady state without stimulation, indicating considerably similar gene expression patterns between them. In addition, DEG analysis of SKM-G4S-28z and SKM-W218-28z CAR-T cells extracted only 16 genes with statistical significance, which have not been previously reported to be associated with T cell exhaustion. This was in sharp contrast to the DEGs between SKM-G4S-28z and SKM-G4S-BBz CAR-T cells and those between SKM-W218-28z and SKM-W218-BBz CAR-T cells, with the same criteria up to 697 and 836, respectively (data not shown). Nevertheless, notably, IL-5 was extracted as the top DEG by q-value among the 16 DEGs between SKM-G4S-28z and SKM-W218-28z CAR-T cells (**Table 1**) because secretion of IL-5 was also upregulated in stimulated SKM-W218-28z CAR-T cells. IL-5 is a Th2 cytokine that induces B-cell and eosinophil activation in mice and humans, respectively (16). Although little is known about the role of IL-5 in CAR-T cells or cytotoxic T cells, it has been reported in a mouse model that immunization with an oxidized mannan MUC1 fusion protein results in the production of IL-5 by Tc1 type T cells, which contribute to the cytotoxic response to tumors (17). In addition to these qualitative changes, there may be changes in the magnitude or kinetics of the CAR signals. Further studies are required to address these issues.

Despite the improved *in vitro* killing capabilities comparable to SKM-G4S-BBz CAR-T cells, SKM-W218-28z CAR-T cells did not show tumor control *in vivo*. This discrepancy may be due to the very slow growth of ACC-MESO4 cells in NSG mice (**Figure 7**), where longer persistence of CAR-T cells in mice would be important for tumor control. We showed that substitution of the G4S linker with the Whitlow/218 linker did not affect the *in vitro* exhaustion status of CAR-T cells (**Figure 3**), possibly because there was no change in the extent of self-aggregation (**Figure 2**), but that it did affect cytokine secretion after tumor encounter (**Figure 5C**). In addition, the upregulation of PD-1 and LAG3 after tumor encounter was more pronounced in SKM-W218-28z CAR-T cells than in SKM-G4A-28z cells (**Supplementary Figure S5**). These changes caused by antigen-induced CAR signaling may be related to improved short-term killing activity, which contributes to *in vitro* assays, but not to long-term persistence of CAR-T cells, which may be important for *in vivo* tumor cell control. Indeed, at the endpoint of the mouse experiment, we did not detect any CAR-T cells in the tumors or spleens of SKM-W218-28z or SKM-G4S-28z CAR-T treated mice. In addition, it is possible that the treatment dose of CAR T cells (1x10^6^ cells/mouse) was not sufficient for *in vivo* tumor control by SKM-W218-28z CAR-T cells, although we used the same treatment dose as in our previous study where we were able to demonstrate the difference in *in vivo* tumor control between SKM-G4S-28z and SKM-G4S-BBz CAR-T cells (4). Therefore, higher doses of CAR-T cells may be recommended in future experiments. Alternatively, the *in vivo* tumor model used in this study may not be appropriate for evaluating the *in vivo* functions of CAR-T cells, because the immunodeficient NSG mice used for tumor implantation may lack sufficient immune molecules and environment to induce and/or maintain the *in vivo* functions of human CAR-T cells. Indeed, a similar discrepancy between *in vitro* tumor cell killing and *in vivo* tumor control has been reported with other self-activating CAR-T cells (1, 2).

The present study focused on the modification of SKM-CAR, which was found to be a self-activating form with an exhaustive phenotype in our previous work. CAR constructs with similar phenotypes have been reported by others, such as GD2-CAR (1) and cMet-CAR (2). Confirming our observations of SKM-CAR with these CARs would increase the generalizability of our findings, but this is beyond the scope of the present study and is a future goal in the field.

In conclusion, we compared the G4S and Whitlow/218 linkers in self-activating SKM-CAR-T cells. Conversion of the G4S to the Whitlow/218 linker did not alter cell surface phenotypes, NFAT and NFκB signaling intensities, and gene expression profiles in CAR-T cells. On the other hand, conversion of G4S to the Whitlow/218 linker significantly altered cytokine expression in stimulated CAR-T cells and improved the *in vitro* killing activity of SKM-28z CAR-T cells to the level of SKM-BBz CAR-T cells, but not *in vivo* tumor control. Although the current study did not reveal the detailed molecular mechanisms underlying these effects, this is the first report describing the advantages of the Whitlow/218 linker over the G4S linker in some aspects of CAR-T cell functions.

## Data Availability

The datasets presented in this study can be found in online repositories. The names of the repository/repositories and accession number(s) can be found below: https://www.ncbi.nlm.nih.gov/geo/, GSE276932.
