# Supplementary figures and images for: Improvement in the function of self-activating chimeric antigen receptor by replacing the linker sequence
Source: Front Immunol. 2025 Apr 16;16:1502607. doi: 10.3389/fimmu.2025.1502607 (PMC12040954; doi:10.3389/fimmu.2025.1502607)

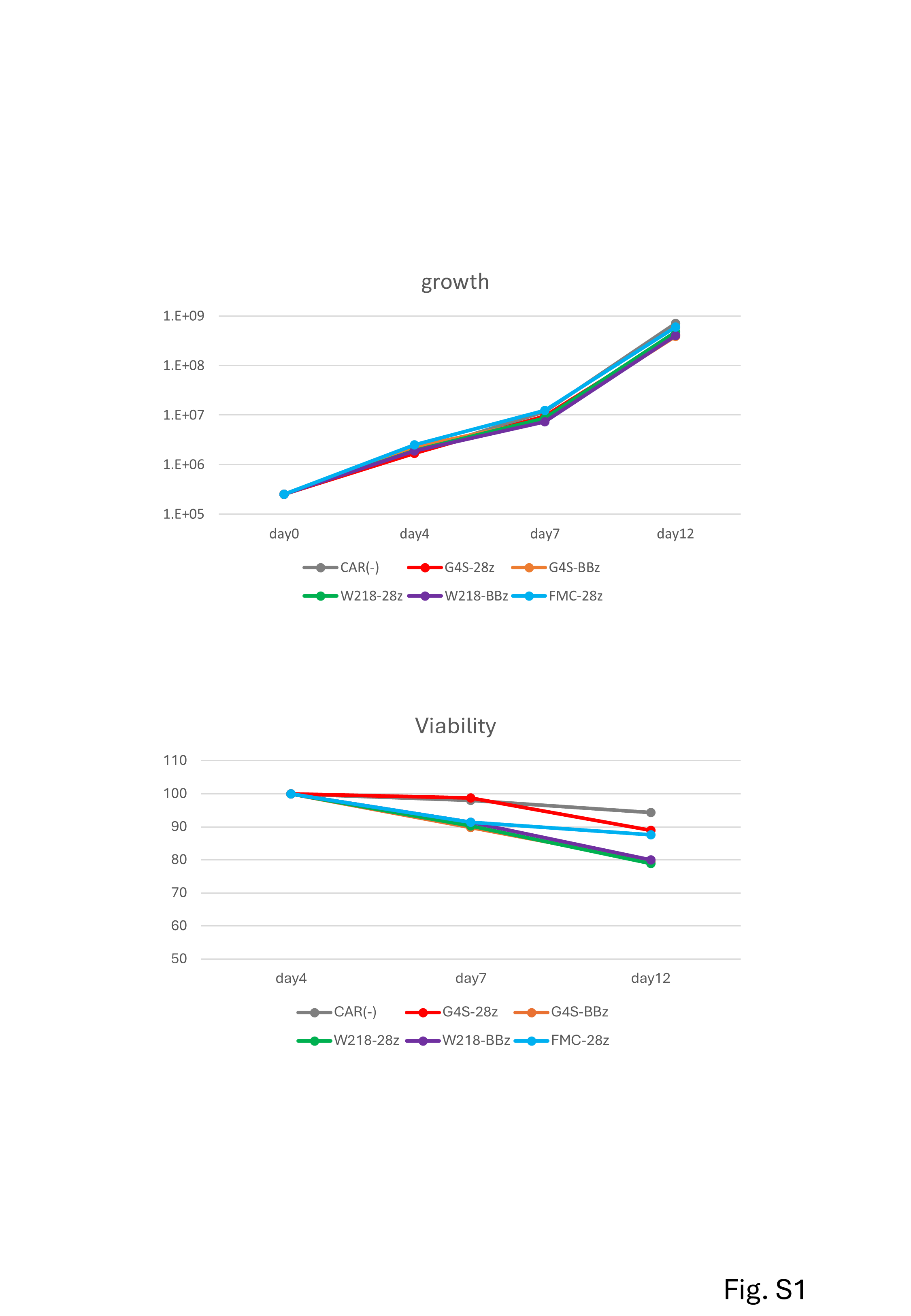

Supplement: Supplementary file 1 [file Image1.tif]

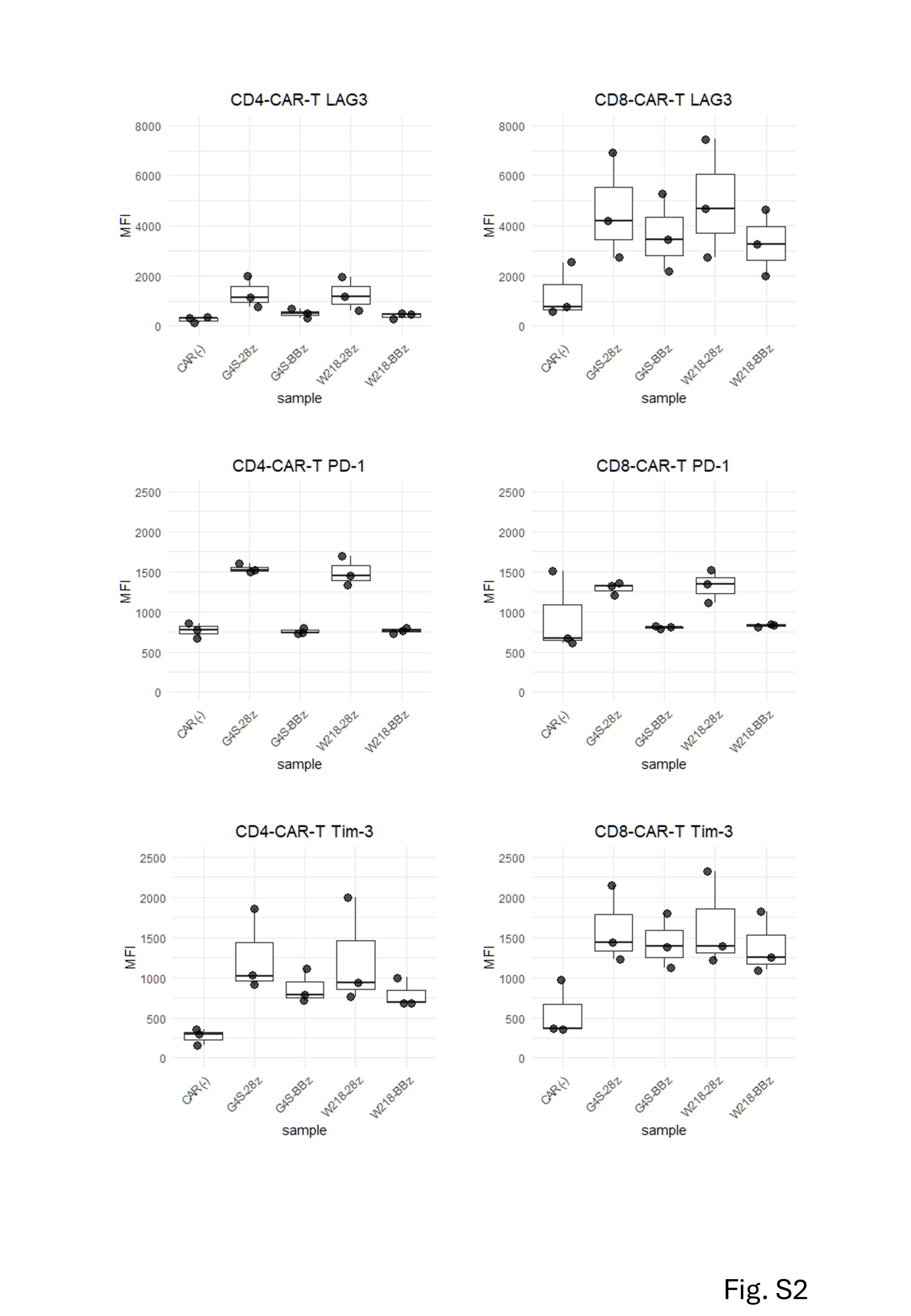

Supplement: Supplementary file 2 [file Image2.tif]

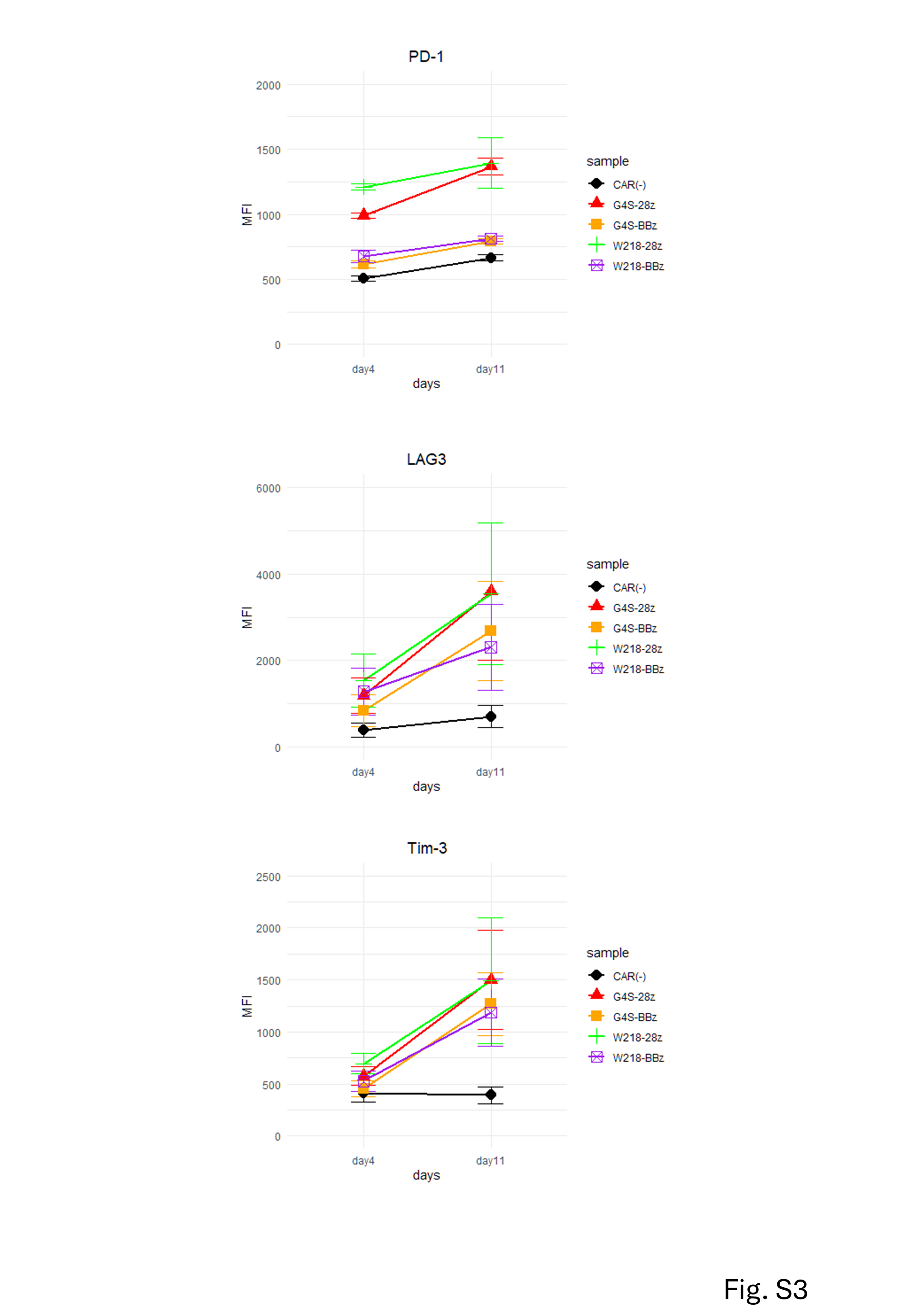

Supplement: Supplementary file 3 [file Image3.tif]

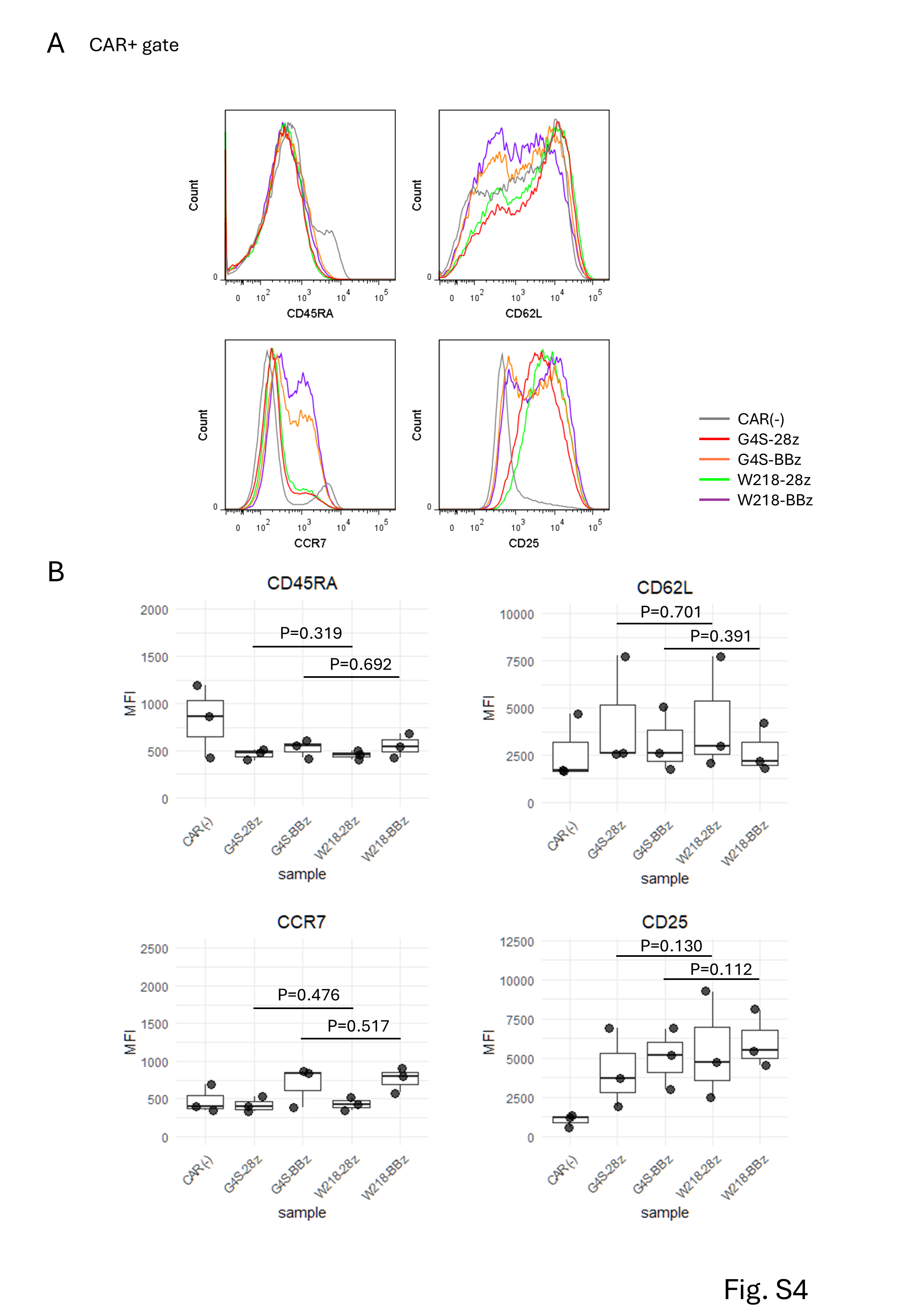

Supplement: Supplementary file 4 [file Image4.tif]

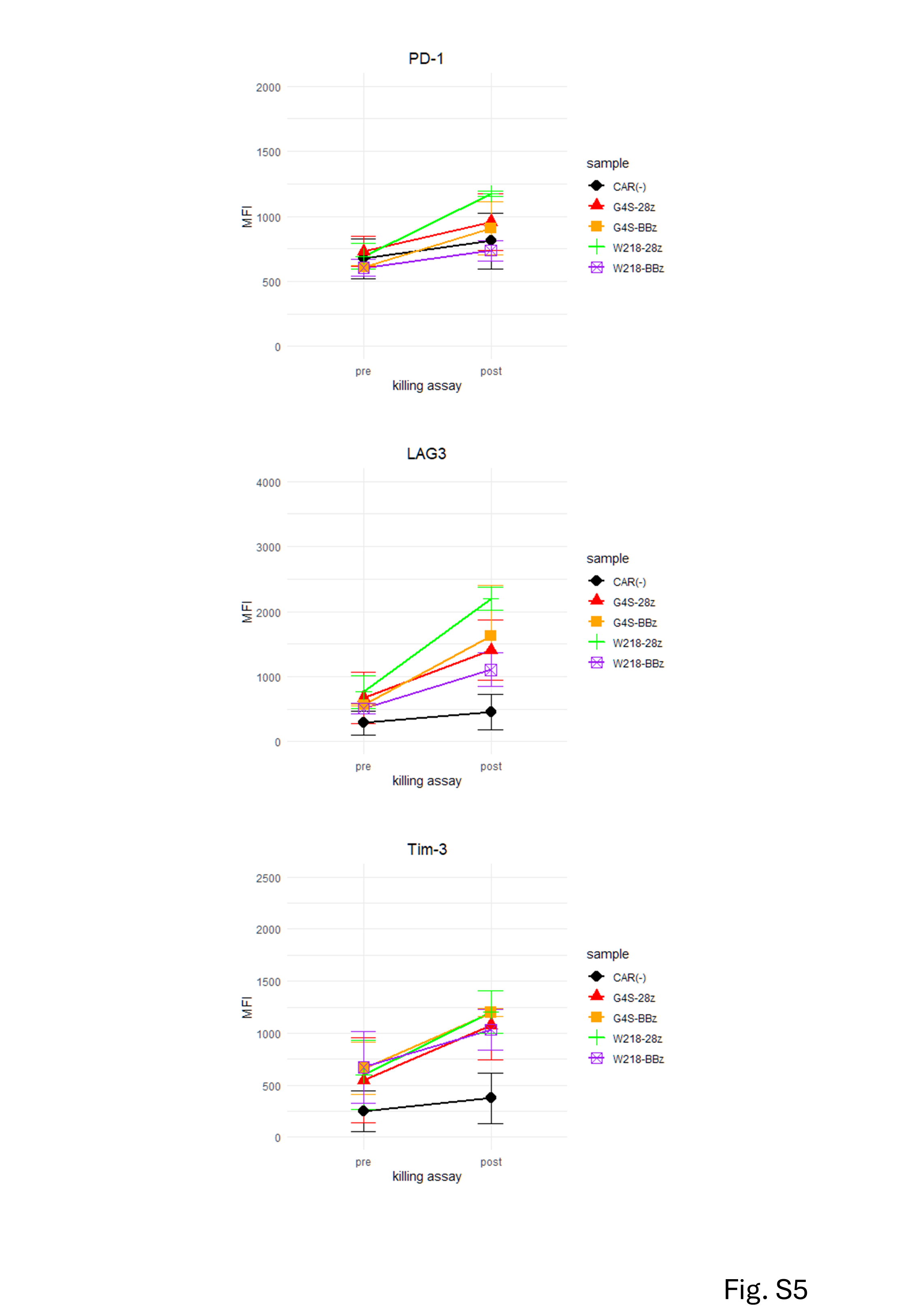

Supplement: Supplementary file 5 [file Image5.tif]
