# Supplementary material for: Improvement in the function of self-activating chimeric antigen receptor by replacing the linker sequence
Source: Front Immunol. 2025 Apr 16;16:1502607. doi: 10.3389/fimmu.2025.1502607 (PMC12040954; doi:10.3389/fimmu.2025.1502607)
Supplement: Supplementary file 6 [file DataSheet1.docx]

Supplementary figure legends

Fig. S1 Ex vivo growth of each CAR-T cells.

CAR-T cells were counted when cultures were diluted and expected total cell numbers were calculated (Upper panel). Upon cell counting, viability of each culture was assessed by trypan blue dye exclusion and plotted (Lower panel).

Fig. S2 Expression of exhaustion markers on CD4^+^- and CD8^+^-CAR-T cells.

FACS data of Fig. 3 were gated into CD8^+^ and CD8^-^ CAR-T cells and MFI of LAG3, PD-1 and Tim-3 were plotted as box plot with individual data point.

Fig. S3 Kinetics of exhaustion marker expression.

MFI of exhaustion markers on day 4 and day 11 CAR-T cells were shown as line plots. Error bar represents SD of three independent experiments.

Fig. S4 Expression of T cell activation and differentiation markers.

CAR-T cells at day 11 from retrovirus infection were stained for surface molecules as shown. A. representative histogram of each marker on CAR+ fraction. B. MFI of each marker in the CAR^+^ fraction from two independent experiments are shown as box plots with individual data point.

Fig. S5 Phenotype of CAR-T cells after target cell killing.

MFI of exhaustion markers before and after killing assay are shown. Error bar represents variation of two independent experiments.
